# Promoting economic and environmental resilience in the post-COVID-19 era through the city and regional on-road fuel sustainability development

**DOI:** 10.1038/s42949-022-00078-6

**Published:** 2022-12-15

**Authors:** Chuxiao Yang, Haitao Wu, Yunxia Guo, Yu Hao, Zhaohua Wang

**Affiliations:** 1 School of Management and Economics, Beijing Institute of Technology, Beijing, 100081 China; 2 Center for Energy and Environmental Policy Research, Beijing Institute of Technology, Beijing, 100081 China; 3 Yangtze River Delta Region Academy of Beijing Institute of Technology, Jiaxing, 314001 China; 4 Sustainable Development Research Institute for Economy and Society of Beijing, Beijing, 100081 China; 5 Beijing Key Lab of Energy Economics and Environmental Management, Beijing, 100081 China; 6 Research Center for Sustainable Development & Intelligent Decision, Beijing Institute of Technology, Beijing, China

**Keywords:** Energy economics, Economics, Energy policy

## Abstract

How to control the global temperature rise within 1.5 °C in the post-COVID-19 era has attracted attention. Road transport accounts for nearly a quarter of global CO_2_ emissions, and the related sulfur dioxide (SO_2_) emissions also trigger air pollution issues in population-intensive cities and areas. Many cities and states have announced a timetable for phasing out urban-based fossil fuel vehicles. By combining a Markov-chain model with a dynamic stochastic general equilibrium (DSGE) model, the impacts of on-road energy structural change led by phasing out fossil fuel vehicles in the road transportation sector are evaluated. The impact of automobile emissions (both CO_2_ and SO_2_) on the environment is evaluated, taking into consideration of variation between cities, regions, and countries. Two other major driving forces in addition to CO_2_ emissions reduction in promoting fossil fuel vehicles’ transition toward net-zero carbon are identified and analyzed with multiple different indicators. Under the framework of the DSGE model, climate policy instruments’ effects on economic development, energy consumption, and their link to economic and environmental resilience are evaluated under exogenous shocks as well.

## Introduction

United Nations Secretary-General António Guterres recently called for stronger ambitions in nationally determined contributions (NDCs) around the world in the 26th Conference of the Parties (COP26). Limiting global average temperature increases to 1.5 degrees requires a 45% reduction in carbon dioxide (CO_2_) emissions in 2030, according to the Intergovernmental Panel on Climate Change (IPCC). Among all sectors, transport typically accounts for the majority of oil demand and is closely related to air pollution issues in population-intensive cities and areas^1^. Since transportation emissions contribute to about 23% of total energy-related CO_2_ emissions worldwide^2^ and approximately 72% from road transport^3^, more than twelve countries planned to phase out fossil fuel vehicles and eventually banned their sales^4^ for transport sector decarbonization. Although transport demand has significantly decreased since the outbreak of COVID-19 and its demand drop contributed the largest share to the decline in 2020 emissions^5^, the recovery of global transport activity is an important bellwether for the rebound in global oil demand and in global CO_2_ emissions^6,7^.

The concept of banning the sale of gasoline vehicles was first proposed by the Netherlands and is scheduled to be fully implemented in 2025. Since then, Ireland, Slovenia, Israel, France, Sri Lanka, the United Kingdom, Sweden, and China have all announced plans to ban the sale of gasoline vehicles^8–11^. Under these circumstances, the Ministry of Industry and Information Technology (MIIT) announced that China will support cities and regions to become pilots for the banning sale of fossil energy vehicles when the conditions are sufficient. This is consistent with the requirement of 30–60 dual carbon goals. China’s transport sector is responsible for ~10% of national CO_2_ emissions^12^, and it is also a substantial source of air pollution^4,13^. To alleviate the cities’ pressure on energy and the environment and promote the upgrading of the automobile industry, it is imperative to ban the sale of gasoline vehicles^14^. In addition, oil consumption in China is highly dependent on imports, while geopolitical risks (GPRs) may lead to safety issues and uncertainties^15,16^.

However, a retaliatory growth of CO_2_ emissions was found in the post-COVID-19 era through the short- and long-term analysis^7^. A rebound in the consumption of motor gasoline was found in May 2020^17^. Since the emission reductions associated with the pandemic are only temporary, the CO_2_ emissions may skyrocket again in reviving economies. Since China is the first major economy to show recovery after a slowdown induced by the COVID-19 pandemic^18^, hereafter, balancing economic growth with energy use and CO_2_ emissions reduction is still an important issue confronting China.

Carbon neutrality in the post-COVID-19 era requires new pathways and solutions to meet the energy demands previously met by fossil energy^19,20^. In addition, restraining oil consumption is important for countries facing a shortage of oil resources^21,22^ for energy security and environmental protection. Many countries in the world are experiencing a shortage of oil resources and depend on imports, for example, the European Union, Japan, Korea, India, and China. Taking China as an example, external oil dependence has exceeded 70% in three consecutive years. Since oil consumption by vehicle usage accounts for ~60% of total oil consumption in China, from this perspective, the deactivation of fossil fuel vehicles has its own internal driving force for these countries. The decarbonization of the transportation sector requires a massive transformation that involves promoting and increasing renewable shares in the vehicle fuel mix^23^, and electric vehicles are considered to be the main method to solve these problems^24,25^. Solutions to mitigate conventional vehicles’ CO_2_ emissions and SO_2_ emissions in the urban area can be categorized into banning fossil fuel vehicles and promoting electric vehicles and other renewable energy vehicles. These changes, along with investments in complementary infrastructure such as charging stations, make electric vehicles viable substitutes for many modes of transportation in cities and regions. However, the transition to electrification has moved slower than expected, and many potential adverse effects have arisen during this transition to BEVs, generally involving the range, initial investment, and charging equipment insufficiency. Regarding transportation carbon emissions, policy instruments represented by personal carbon trading schemes are still under discussion^26,27^. The costs of these policy instruments remain unknown^28^.

A successful transition toward a net-zero economy and the achievement of carbon neutrality in the transportation sector of population-intensive areas, such as urban areas, require the scaling up of low-carbon vehicle investment and the divestment of carbon-intensive vehicle investments and production^29^. For the transportation sector, banning the sale of fossil fuel vehicles to achieve the upcoming stringent carbon emissions reduction targets is imperative^10^, i.e., carbon peak by 2030 and carbon neutrality by 2060. Since 2005, China has become the world’s largest vehicle-producing country, with nearly 25% of total global vehicle production. Whether it undertakes the common but differentiated responsibilities (CBDR) determined by the Kyoto Agreement or actively responds to the more radical carbon emission reduction target proposed by COP26, cities and regions in China need to pay more attention to the impact of fossil fuel vehicles.

Therefore, we explore the macroeconomic effects of different emission reduction policy instruments in China by applying a static comparison method using both deterministic and stochastic general equilibrium models. Based on the analysis of road transport energy demand, related emissions, and its impact on economic development and resilience, we also compare the related emissions with those of other countries, such as the United States, Japan, the European Union, and India. Although the notion of “resilience” was first used in physical, engineering, and organizational science, it has recently gained popularity in economics and regional analysis^30^, especially since the outbreak of COVID-19. Most research focuses on the resilience of cities and entrepreneurs. For example, Hardaker et al. studied the resilience of city retailers under the shock of COVID-19^31^. Cheng et al. evaluated Chinese cities’ resilience in terms of spatial-temporal evolution^32^.

Additionally, we explore the role of different policy instruments under the deterministic economy and with consideration of exogenous stochastic shocks in the future through a dynamic stochastic general equilibrium model. Such an integrated strategy considering the demands in fossil fuel vehicles and their related energy consumption, especially the shortage of oil resources in many countries represented by China, can be viewed as an early entry point to address carbon emission mitigation, sulfur dioxide emissions impact on the environment quality and human health in population-intensive urban areas, and national energy security challenges.

## Results

### Demand analysis by transport mode and vehicle powertrain

The types of transport modes among cities and regions can be categorized into four different uses, such as railways, roads, waterways, and civil aviation. To clarify the significance of fossil energy, especially fuel oil demand, we use the Markov-Chain prediction model to predict how transport demand will change in the future from the perspective of the rotation volume of both freight and passengers.

According to the results, a significant change in road transport has occurred since the outbreak of the COVID-19 pandemic. An unusual decreasing trend in the rotation volume of both freight and passengers in road transport and a significant increase in railway volume indicates that people prefer to avoid road use and choose railway travel instead. Given the significant decrease in the total volume of both passengers and shipments, we can infer that people prefer to stay at home instead of travel compared to the years before COVID-19^6^, conducting trips only if they are necessary (visiting parents during the spring festival, for example). The proportion of convenient and efficient travel is increasing. This is also a representation of the policy launched in the 14th Five-Year Plan for National Economic and Social Development and Vision 2035 of the People’s Republic of China about road transition to railway and waterway.

**Fig. 1:**
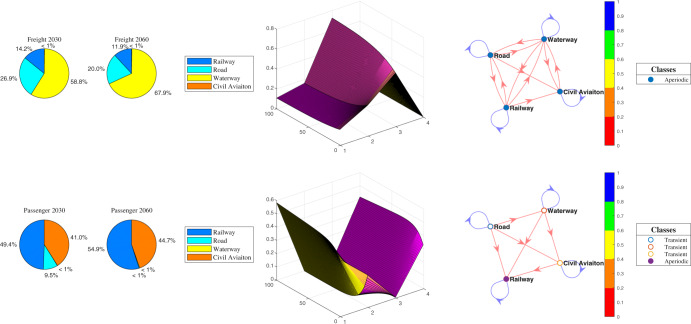
Forecasting ratio to transport mode by end-use. Figure 1 depicts the forecasting results of the four types of transport mode probability transition processes (i.e., railway, road, waterway, and civil aviation) from 2030 to 2060, categorized by end-use. The pie charts (first and second column) depict the predicted share of each type of demand of transport mode to the total volume of turnover in 2030 and 2060. Partial future demand in civil aviation (freight transport) and waterway is not clearly shown in the pie chart because their share is under 1%. The fourth column depicts the visualization of the average probability transition matrix corresponding to each type of rotation volume, where the x-axis and y-axis denote four states and each state’s transition probability, respectively. The fifth column depicts the visualization of the discrete-time Markov-chain state transition matrix. The higher the transition probability, namely heading to 1, the color closer to blue. The smaller the transition probability, the color closer to red.

Although transport demand has significantly decreased since the outbreak of COVID-19 and its demand drop contributed the largest share to the decline in 2020 emissions^5^, the recovery of global transport activity is an important bellwether for the rebound in global oil demand and in global CO_2_ emissions^33^. In the future, the long-term fluctuation of the epidemic and the overall trend of economic growth after the epidemic are expected to coexist for a long time Fig. 1. This also shows the inseparable and interrelated relationship between transportation demand, travel activities, and economic growth.

**Fig. 2:**
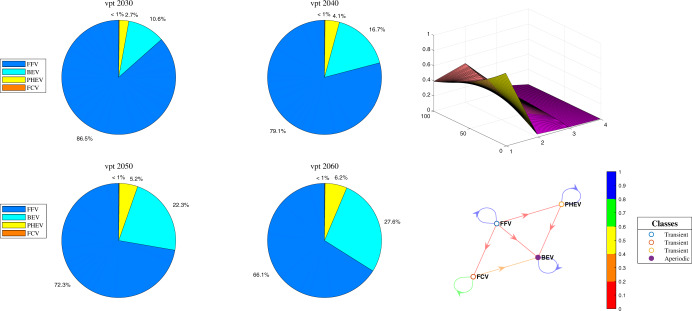
Forecasting ratio of vehicle consumption by vehicle powertrain. Figure 2 depicts the forecasting results of the four types of vehicle consumption for the total sale of vehicles (i.e., FFVs, BEVs, PHEVs, and FCVs) from 2030 to 2060, categorized by powertrain type. The vehicle powertrain is denoted by “vpt”. The pie charts (col. 1 and 2) depict the consumption ratio of each type of powertrain to the total sales of vehicles from 2030 to 2060. Partial future demand for FCVs is not clearly shown in the pie charts because their share is under 1%. In the third column, the upper right subplot depicts the visualization of the average probability transition matrix corresponding to each type of powertrain, where the x-axis and y-axis denote four states and each state’s transition probability, respectively. And the lower right subplot depicts the visualization of the state transition matrix. The higher the transition probability, namely heading to 1, the color closer to blue. The smaller the transition probability, the color closer to red.

Based on the types of powertrain, vehicles can be categorized into conventional fossil fuel vehicles (FFVs) and new energy vehicles (NEVs). NEVs can be divided into three different types, namely, battery electrical vehicles (BEVs), plug-in hybrid electrical vehicles (PHEVs), and fuel cell vehicles (FCVs), by the accessibility of data. The period of the dataset is from 2013 to 2018. We can predict each type of vehicle consumption ratio to the total vehicle consumption from 2030 to 2060 by applying a Markov-chain forecasting model. The predicted results are depicted at a 10-year interval in Fig. 2.

The results show that the ratio of FFVs and NEVs generally decreases and increases, respectively. Among the NEVs, BEV and PHEV demand increases smoothly, while the share of FCVs accounts is too small to play an important role in vehicle transition in the next four decades. The increase in NEVs is connected to the stimulus policies launched by the Chinese government in recent years. By employing subsidies in purchasing NEVs and investing in the charging station, the two most-watched concerns of consumers in choosing NEVs instead of FFVs are being eliminated.

According to the estimation results of Lin and Du^34^, the share of the transportation sector in urban areas accounts for ~60% of the total consumption of crude oil. Based on the Markov-chain forecast model, we can predict that demand in FFVs will be ~87% and 66% in 2030 and 2060, respectively. Compared to the level in 2020, the corresponding decrease amount in terms of the total sales of vehicles is ~8.09% and 31.53%. Using the energy consumption inventory data of CEADs, we can calculate the total CO_2_ emissions reduction by the structural change in vehicles in terms of the powertrain.

### Energy shortage in transport becomes a driving force

The additional transport fuel consumption mainly comes from demand in the transportation sector. Although China has employed a state-centered approach toward energy security, which has led to cooperation with its neighboring countries, the sophisticated bi- or multilateral political relationship faces considerable uncertainty.

China became the largest importer of oil–gas resources in the world in 2019. Since 2015, the additional consumption of the raw material of transport fuel has depended entirely on imports. It is centralized in comparison to coal, which is commonly used for power generation. Oil is frequently used as automobile fuel, and its consumption patterns are decentralized. And it is strongly related to transportation and other domestic consumption fields, so the policy application points are different. Although the ratio of new energy vehicles in the car market has grown dramatically in recent years^35^, it accounted for only 5.4% in 2020. Therefore, fossil fuels in the transportation sector still play an important role, which is related to consumption habits^36,37^.

In addition, China is highly dependent on oil imports, generating potential risk because each country in the international market competes with each other. The Markov-based energy prediction model can capture this shared competition when the sum of all the participants equals one.

Based on BP energy statistical data from 2020, the main importers of oil in the world, such as China, the United States, Europe, India, and Japan, are considered. For the period 2010 to 2019, the share of each country or region in each year can be calculated. Based on the prediction results of the Markov-based model, the future import share of the countries and regions mentioned above can be obtained. In contrast to other linear forecasting methods, the Markov-chain-based model can capture competition in the share of imports among importers in the global market. Assuming no major change in global total oil exports and relatively steady growth in the economy, the predicted share of raw material for transport fuel imports for China would be 42.7% in 2030 when there are no exogenous constraints. However, this increasing share on the demand side cannot be satisfied by the international oil market, given geopolitical risk and supply capacity.

Therefore, triggering a structural transition from fossil fuel vehicles toward new energy vehicles, especially for the urban areas with intensive populations and a large amount of private car possession, is of great benefit to alleviate this potential imbalance between supply and demand in fuel oil. In other words, the imbalance between supply and demand in the future can also become an internal driving force for phasing out fossil fuel vehicles.

### The variation among cities and regions for CO_2_ emissions of fossil fuel vehicles

In fact, the initial driving force for countries that brought up phasing out FFVs is to reduce carbon dioxide emissions. CO_2_ emissions of the transportation sector account for the second rank of the total emissions for a country, but its impact has a wide range and involves consumption habits. Since China is a country with a large population and a vast territory, variations between cities and regions become a significant factor in analyzing energy transitions in the transportation sector. To date, the majority of previous studies have focused on the country level rather than the possible urban or regional impact, and the key point in reducing transport emissions remains unknown. To determine the importance of phasing out fossil fuel vehicles with the consideration of regional heterogeneity, using regional microdata to analyze these issues is important and helpful in improving the accuracy of policy instrument designations.

**Fig. 3:**
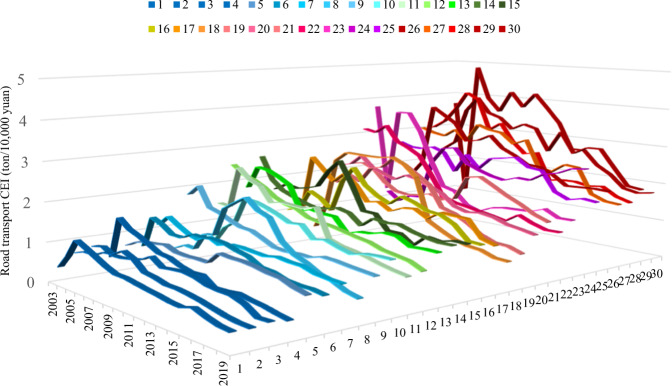
Cities (and provincial) carbon intensity of road transport in China. Figure 3 depicts the results of the cities and provincial carbon emission intensity of China’s transportation sector from 2003 to 2019. The numbers from 1 to 30 denote thirty cities or provinces in China in the constraint of data accessibility. Please refer to Supplementary Table 1 in the supplementary information file to obtain the corresponding name of each city or province represented as numbers in Fig. 3.

**Fig. 4:**
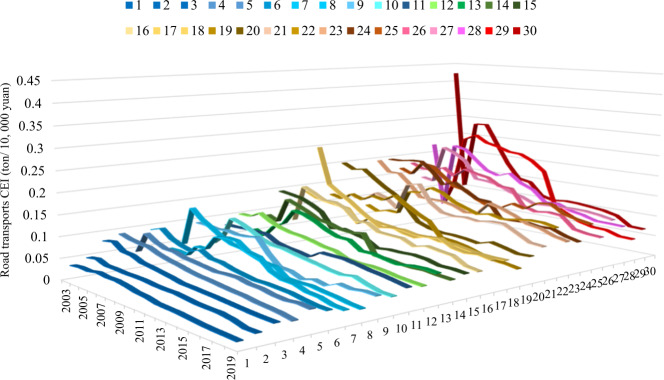
Cites and provincial CEI of road transportation in China. Figure 4 depicts road transportation-related CEI by the transportation sector’s GDP rather than the total GDP of China. The x-axis and y-axis represent the 30 cities or provinces and year, respectively. The z-axis represents the carbon intensity of road transportation. Please refer to Supplementary Table 2 in the Supplementary Information file to obtain the corresponding name of each city or province represented as a number in Fig. 4.

Both Figs. 3 and 4 depict the carbon emissions intensity of cities’ and provinces’ transportation sectors. These two figures, however, are calculated using different methodologies. In Fig. 3, the road transportation emissions are divided by the GDP of each province or city. In Fig. 4, the road transportation emissions are divided by the transportation sector’s GDP. A significantly higher deviation and fluctuation are shown in Fig. 3 than in Fig. 4. The original data resources come from the CEADs database, which contains cities and provinces’ energy inventory from 2003 to 2019. The energy inventory data of each year contain 30 sheets corresponding to 30 cities and provinces in China in constraint of data accessibility.

Figure 3 presents an overview of cities’ and provinces’ carbon emission intensity (CEI) of road transport in China from 2003 to 2019. As shown, a pronounced discrepancy in the level of CEI is represented among cities and regions. During this period, the three provinces with the highest CEI values were Yunnan, Xinjiang, and Guangxi; their average CEI values reached as high as 3.011, 2.897, and 2.649 tons per ten thousand yuan, respectively. The three cities or provinces with the lowest CEI values were Hebei, Beijing, and Shanghai, with average CEI values equal to 0.671, 0.701, and 0.742 tons per ten thousand yuan, respectively. When using the GDP of the corresponding province or city as the denominator for the road transport CEI calculation, however, given the comparatively lower level of economic development in Yunnan, Xinjiang, and Guizhou, these three provinces become the regions with the highest CEI but with a lower level of private car ownership.

Furthermore, we use the GDP of the transportation sector as the denominator for the road transport CEI, and the results are shown in Fig. 4. Using this method, the three provinces with the highest average CEI values are Yunnan, Inner Mongolia, and Ningxia, which indicates that these three provinces need to consume more energy and transport capacity per unit GDP. This is also indicated that these three cities or provinces’ economic benefits of the transportation sector is relatively low. In addition, Ningxia, Yunnan, and Inner Mongolia belong to a sparsely populated area in China. The differences between vast land and sparse population or road mileage may cause these three places to cost more energy to reach amenities than in a dense city.

From the perspective of promoting cities and provincial economic development, the road transport CEI reflects a significant regional heterogeneity in energy efficiency regardless of the direct effect or indirect effect. Furthermore, banning the sale of fossil fuel vehicles in cities and provinces may generate greater impacts for regions with comparatively lower economic development levels. Therefore, variation among cities and regions must be considered during the designation of policy instruments for promoting the transition to transportation mode by phasing out fossil fuel vehicles.

### Analysis of SO_2_ emissions intensity of fossil fuel vehicles in cities and regions

Different from the impact of CO_2_ emissions on global climate change, the pollutant emissions represented by sulfur dioxide (SO_2_) generated from fossil fuel vehicles mainly affect the environmental quality and resident health of cities and their surrounding areas. In the existing literature, SO_2_ per capita is commonly used as the SO_2_ emission intensity, especially at the regional or provincial level, for comparison.

**Fig. 5:**
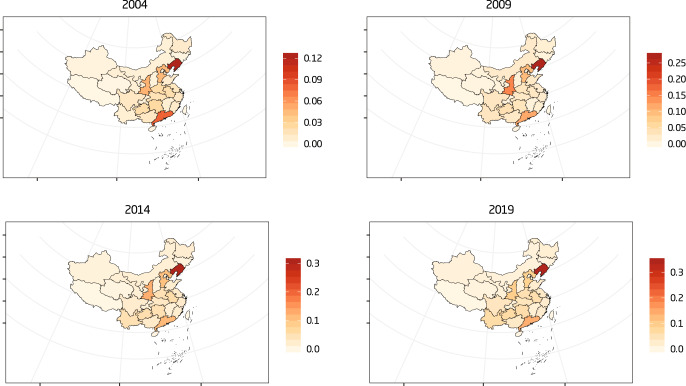
Regional road transport SO_2_ emissions intensity trends. Figure 5 depicts the results of trends in cities and provincial SO_2_ emissions from road transportation from 2004 to 2019. The indicator used here is sulfur dioxide concentration measured by SO_2_ emissions per capita. The unit is tons per 10 thousand people.

Figure 5 depicts the trends of cities and provincial SO_2_ emissions per capita from 2004 to 2019 every five years. From the perspective of comparison, a significant increase in the SO_2_ emissions intensity has been shown across the 30 provinces in China. In 2009, the limitation of the concentration was less than 0.05 tons per ten thousand people, while in 2019, this limitation was increased to 0.16 tons per ten thousand people. In 2003, there were three provinces with SO_2_ concentrations higher than 0.01 tons per ten thousand people: Hebei, Guangdong, and Guangxi Zhuang Autonomous Region. In 2019, there were 16 provinces with SO_2_ intensity higher than 0.01 tons per ten thousand people, and two of them were higher even than 0.05 tons per ten thousand people. Further analysis indicates that this increasing trend results from local economic growth and a rapidly increasing amount of total vehicle ownership. Fossil fuel vehicles account for the majority of the total vehicle ownership, especially in urban-based areas. SO_2_ emissions can have a negative impact on local residents’ health conditions in cities or provinces, and improve the corresponding health cost.

**Fig. 6:**
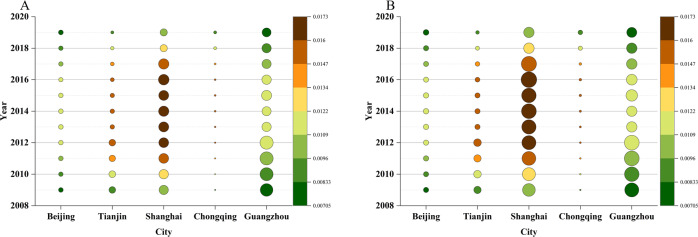
Regional roadway sulfur dioxide emission intensity in typical cities. The denominator is the roadway length (**A**) and the unit area of the administrative division (**B**). Five different typical cities are chosen as samples considering their economic development level and vehicle ownership. The size of each bubble represents the level of SO_2_ emissions intensity.

**Fig. 7:**
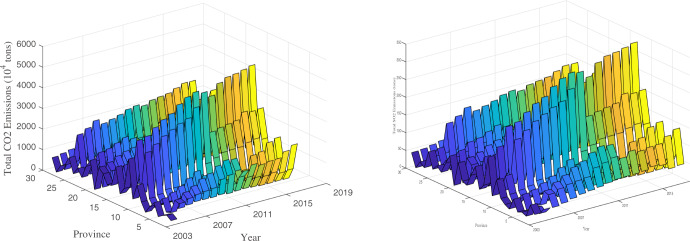
Urban and provincial road transport CO_2_ and SO_2_ emissions. Figure 7 depicts a distribution trend in total road transportation of CO_2_ (on the left) and SO_2_ (on the right), respectively. The x-axis denotes the time period from 2003 to 2019. The y-axis denotes 30 cities and provinces of China by the constraint of data accessibility. The original resource of road transport energy consumption is the energy inventory list in the CEADs database. Please refer to Supplementary Table 3 in the Supplementary Information file to obtain the corresponding name of each city or province represented as a number in Fig. 7.

Considering that the sulfur dioxide emissions of road transportation are more concentrated in the central urban areas, the SO_2_ intensity can be better represented by emissions per unit road mileage and emissions per unit area. Therefore, we calculated and compared the SO_2_ emissions per unit road mileage and unit area in five of the top largest private car-owned cities in China to obtain the pollution impact of sulfur dioxide emissions on central cities. The results are shown in Figs. 6 and 7.

In fact, road transport SO_2_ emissions are generated when fossil fuel vehicles run on roadways^38,39^. It lacks realistic meaning to use the total population or private car ownership as the denominator to calculate the SO_2_ intensity. Therefore, a rational indicator to represent road transport SO_2_ emissions is of significant importance. As shown in Fig. 6, the road transport SO_2_ intensity is calculated using the total roadway mileage (on the left) and the total land area of the administrative division (on the right) as the denominator. Both subfigures show a similar trend for each city over time, which indicates that using these two variables as the denominator to obtain the road transport SO_2_ intensity is rational. When referring to the size of the bubble, Shanghai and Guangzhou have the largest SO_2_ intensity compared to the other three cities. For each city, the road transport SO_2_ intensity changes little over time, which indicates a sophisticated connection among the economic growth rate, roadway construction, urban expansion, and FFVs increase. This five-city sample can clearly reflect the sophisticated connections among these variables.

Compared to the initial driving force of banning the sale of FFVs is CO_2_ emissions reduction, the reduction of SO_2_ emissions generated by FFVs would become big cities’ driving force of phasing out FFVs, especially for those with a large amount of private vehicle ownership. This is because SO_2_ emissions would exacerbate people’s health conditions and lower the surrounding environmental quality in resident-density areas. These negative impacts cannot be eliminated by the three-way catalytic converter, which focuses on converting NO_x_, C_n_H_m_, and CO into harmless and nontoxic N_2_, H_2_O, and CO_2_. In addition, sulfur-containing automobile exhaust may damage TWC converters and poison them. This negative effect can increase the emissions of NO_x_, CO, and C_n_H_m_ if the TWC converter becomes invalid and further dampen the environmental quality. On the other hand, the usual technical in SO_2_ emission reduction is to decrease its share during the processing period, and it is common to reach the level of less than 10 μg per gram (the level of element sulfur <10 μg per gram). However, any further increase in the decreased degree would increase the production cost and be technically difficult to achieve. From the perspective of economic development, however, the improvement in the resident living level is bound to prospectively increase the ownership of private vehicles. Hence, a phase-out of FFVs and a ban on the sale of them eventually would be the reasonable choice for solving the dilemma confronted by us, especially for large cities and metropolises. In addition, the constraint on SO_2_ emissions would become an important grasp for these cities and areas to fulfill the transition in transport mode from FFVs.

### Urban and regional road transport emissions and economic growth

In fact, urban and regional road transport emissions have a close connection with economic development. First, we analyze the total road transport CO_2_ and SO_2_ emissions in 30 cities and provinces of China. Trends in both emissions show an increasing trend over time, which is consistent with empirical evidence from the urban and provincial levels.

A distribution trend in regional (30 cities and provinces) total road transport CO_2_ emissions and SO_2_ emissions from 2003 to 2019 in China (see Fig. 7) shows a similar trend over time. Along the x-axis, the general trend of all 30 cities and provinces shows an upward trend over time. This is consistent with the trend in regional economic development.

We use the static comparison method and rearrange the expression of each variable by substitution into the terms of share to the total output and labor supply in terms of the labor–leisure ratio (*d*_*t*_) as follows.

1 $$k_t \equiv \frac{{K_t}}{{Y_t}} = \frac{{\alpha \left( {1 + \hat \phi _tA_{t,Y}} \right)}}{{\beta E_t\left( {\frac{{C_t}}{{C_{t - 1}}}} \right) - \left( {1 - \delta } \right)}}$$

2 $$m_t \equiv \frac{{M_t}}{{Y_t}} = \frac{{\gamma \left( {1 + \hat \phi A_{t,Y}} \right)}}{{1 + \hat \phi _t}}$$

3 $$c_t \equiv \frac{{C_t}}{{Y_t}} = 1 - m_t - k_{t + 1}\frac{{Y_{t + 1}}}{{Y_t}} + \left( {1 - \delta } \right)k_t$$

4 $$d_t \equiv \frac{{H_t}}{{1 - H_t}} = \frac{{\left( {1 - \alpha - \gamma } \right)\left( {1 + \hat \phi _tA_{t,Y}} \right)}}{{\eta c_t}}$$

We can obtain the expression of the effective shadow price equation as follows.

5 $$\hat \phi _t = \frac{{\gamma /m_t - 1}}{{1 - A_{t,Y}\gamma /m_t}}$$

Equation (5) denotes that the effective shadow price value is connected with emissions per capita and permits allocations corresponding to each policy scenario.

First, we compare how the steady state changes under each policy scenario by abstract variables from its dynamics and eliminate the time subscript *t*. We assume that $\hat \kappa \equiv 1/\left( {1 + \delta } \right)$ and substitute this equation into Eq. (1). Therefore, the steady state of each variable is given by:

6 $$\begin{array}{l}k = \hat \kappa \alpha \left( {1 + \hat \phi A_Y} \right);\,m = \gamma \displaystyle{\frac{{1 + \hat \phi A_Y}}{{1 + \hat \phi }}};\,c = 1 - m- \delta k;\,d =\displaystyle{ \frac{{1 - \alpha - \gamma }}{{\eta c}}}\left( {1 + \hat \phi A_Y} \right)\end{array}$$

We assume that there are four different policy scenarios and that each of them contains only one policy instrument at a time. Using a static comparative method, we can obtain the steady state of each variable under each policy scenario and further evaluate the policy’s effect on the economy from a deterministic perspective. The four policy scenarios we consider here are no policy, emission intensity, emission tax, and emission cap, which are policy instruments that have received a wide range of attention and discussion.

We assume that there is no-policy scenario, which can be regarded as a benchmark of the following static comparison analysis. Under this circumstance, the budget constraint of emissions no longer exists ($\phi = 0,\hat \phi = 0$). Then, we have $k = \hat \kappa \alpha$, *m* = *γ*, $c = 1 - \gamma - \delta \hat \kappa \alpha$, and $d = 1 - \alpha - \gamma /\eta \left( {1 - \gamma - \delta \hat \kappa \alpha } \right)$. Then, we can solve d to obtain $H = \left( {1 - \alpha - \gamma } \right)/\left[ {\eta \left( {1 - \gamma - \delta \hat \kappa \alpha } \right) + 1 - \alpha - \gamma } \right]$. Solving for the steady state of the total output, we can obtain that $Y = Z^{\frac{1}{{1 - \alpha - \gamma }}}\left( {\hat \kappa \alpha } \right)^{\frac{\alpha }{{1 - \alpha - \gamma }}}\left( \gamma \right)^{\frac{\gamma }{{1 - \alpha - \gamma }}}H$.

It is worth noting that under the benchmark scenario, the steady state of the total output share of consumption, capital stock, labor supply, and emissions intermediate inputs are not connected to the productivity variable or the share of leisure time to the whole day of time.

The second policy scenario we would like to analyze is the emission intensity. Assuming that the intensity target is μ, we can obtain the emission constraint as in *A*(*Y*) = *μY*, *A*_*Y*_ = *μ* = *m*. Then, we have$m = \gamma \left( {1 + \hat \phi \mu } \right)/\left( {1 + \hat \phi } \right) = \mu$, from which we can derive the expression of the shadow price value $\hat \phi$ as follows.

7 $$\hat \phi = \frac{{\gamma - \mu }}{{\mu \left( {1 - \gamma } \right)}}$$

Equation (7) is also invariant to the productivity factor. After rearranging and substituting, we can obtain the steady state expression for capital, labor supply, consumption, and emission intermediate inputs as follows.

8 $$k = \frac{{\hat \kappa \alpha \left( {1 - \mu } \right)}}{{1 - \gamma }};\,c = 1 - \mu - \delta \frac{{\hat \kappa \alpha \left( {1 - \mu } \right)}}{{1 - \gamma }};\,d = \frac{{1 - \alpha - \gamma }}{{\eta \left( {1 - \gamma - \delta \hat \kappa \alpha } \right)}}$$

Under the constraint of an intensity target, the steady-state capital, consumption share to the total output, and steady-state labor–leisure ratio is invariant to the productivity factor.

The third policy scenario we would like to analyze is the emission cap. Since the emission intermediate input we consider here consists of both CO_2_ and SO_2_ emissions, we can intuitively infer that the SO_2_ and CO_2_ emissions are generated from fossil fuel vehicles if the representative agent owns a car for production. With an emission cap, *M* is a constant, and $A\left( Y \right) = \bar M$, which indicates that *A*_*Y*_ = 0. Then, the steady-state capital, labor–leisure ratio, consumption, and emission intermediate input can be expressed as follows.

9 $$k = \hat \kappa \alpha ;\,m = \gamma /\left( {1 + \hat \phi } \right);\,c = 1 - \gamma /\left( {1 + \hat \phi } \right) - \delta \hat \kappa \alpha ;\,d = \left( {1 - \alpha - \gamma } \right)/\eta c$$

It is noted that the steady state of capital is constant and equals the same level as in the no-policy scenario. In addition, the steady-state consumption and labor–leisure ratio are identical to the same level as in the no-policy scenario. All the variables in this circumstance (Eq. (9)) is invariant to the productivity factor, while the effective shadow price of emissions is connected to the productivity factor as follows.

10 $$\hat \phi = \frac{{\gamma Zf - \bar M}}{{\bar M}}$$

In other words, the effective shadow price of emissions increases as long as there is an improvement in the productivity factor. A lift in the level of $\hat \phi$ indicates an increase in the price level of the emission permit and generates an additional cost per unit of emission. Since there is a procyclical trend in the shadow price of emissions, each variable in Eq. (12) shows procyclical or countercyclical trends differ from those of the no-policy scenario or the emission intensity scenario.

Taking road transport emissions as an example, we can intuitively understand the impact of an emission cap on the economy. As mentioned above, many countries have planned to phase out fossil fuel vehicles and eventually ban their sale. Therefore, we assume that part of the emission cap corresponds to the emission level generated by FFVs at the time of banning the sale of FFVs. Assuming that the share of energy consumption by FFVs is *ψ*, if the emission intermediate input corresponds to the total energy consumption in the economy, we can obtain the emissions generated by FFVs to be *ψM*. Therefore, the emission cap scenario is fit into the question of banning the sale of fossil fuel vehicles.

The fourth policy scenario is emission tax, with all the revenues conserved into a lump-sup transfer to the representative consumer. Letting the price of per unit emission remain fixed, we can obtain $\hat \phi = \tau$. Then, we can obtain the steady state capital, labor–leisure ratio, consumption, and emission intermediate input as follows.

11 $$k = \hat \kappa \alpha ;\,m = \gamma /\left( {1 + \tau } \right);\,c = 1 - \gamma /\left( {1 + \tau } \right) - \delta \hat \kappa \alpha ;\,d = \left( {1 - \alpha - \gamma } \right)/\eta c$$

With the emissions price fixed, the labor supply and the total output shares of consumption, capital, and emissions are all invariant to productivity changes, as in the no-policy and intensity target scenarios. For the comparison of the steady state of variables under each policy scenario, please refer to Supplementary table 4 in the Supplementary Information file for more details. To numerically understand the abovementioned analytical analysis, we use the calibration method to set values for all the parameters. For more details on the parameter values, please refer to Supplementary Table 5 for more information. Different from Fischer and Springborn^40^, who selected values of parameters according to postwar economic development in the United States, we select the key parameter values according to China’s economy according to the existing literature.

The comparison results show that there is no difference between emission cap and emission tax when there is no uncertainty. Therefore, we choose energy tax for policy assessment and compare it with the business-as-usual scenario under the exogenous shocks.

**Fig. 8:**
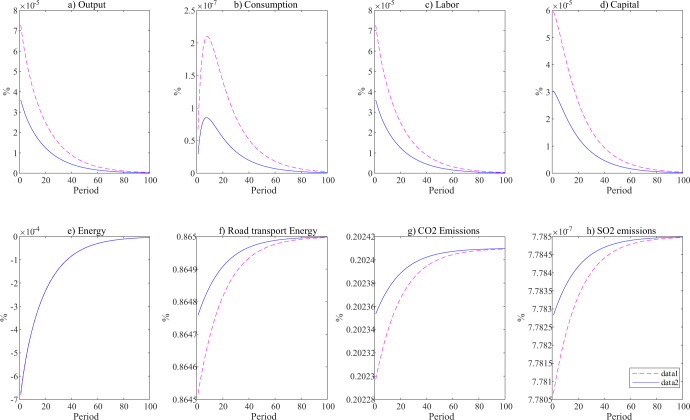
Impulse response of variables to exogenous energy price shock. The two policy scenarios are no policy (red line) and an energy tax (blue line). The x-axis and y-axis represent the time period since the shock happens and the deviation of each variable from their steady state in percentage. All the variables (**a**–**h**) are represented in deviation from their steady state in logarithmic format.

Figure 8 depicts the impulse response of variables to exogenous shocks under both the no-policy (business-as-usual, BAU) and energy tax (ET) scenarios. In general, variables under the ET scenario have a lower growth rate in their corresponding level compared to those under the BAU scenario, which implies that any policy instrument would dampen economic development to some degree. Under the energy tax scenario, the road transport energy consumption fluctuates less than it does under the BAU scenario, which indicates that although a one-unit standard deviation of the energy price shock would decrease each variable in the economy system, since the total energy consumption would decrease when launching an energy tax, the fluctuations would be smoothed correspondingly. Since the total consumption of energy has decreased, the impact of the exogenous shock is smoothed to some degree (compared to the BAU scenario). This is consistent with the direction of expectation. It is reasonable to infer that with the improvement in the substitution rate of new energy vehicles to fossil fuel vehicles, the on-road fuel consumption will decrease, enhancing the ability of the economy to adjust to the effects of adverse unexpected shocks, commonly known as economic resilience.

### Road transport emissions compared to other countries

**Fig. 9:**
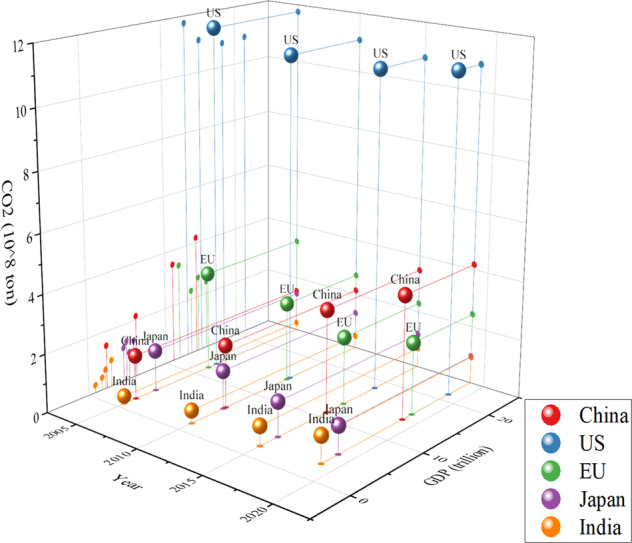
International comparison of road transport CO_2_ emissions. The top five countries and regions in terms of energy consumption are selected as examples for comparison among different countries. The x-axis represents the year. The y-axis represents gross domestic production in trillion US dollars. The z-axis represents the road transport’s CO_2_ emissions.

**Fig. 10:**
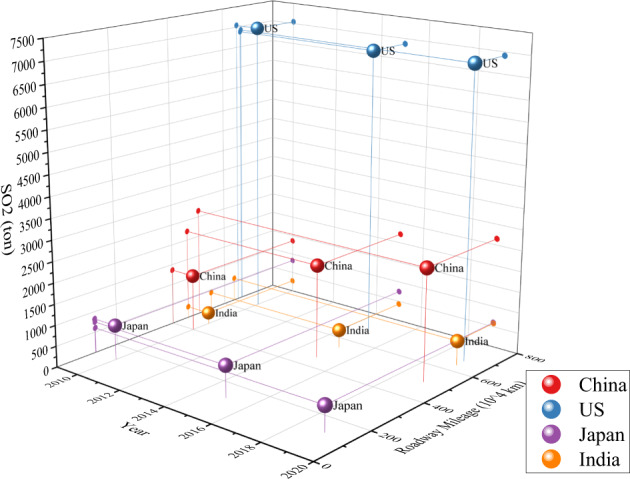
International comparison of road transport SO_2_ emission intensity. The data include the following: SO_2_ emissions, calculated according to the oil consumption used as motor gasoline in each country, and roadway mileage, using public road mileage as the measure. The top four energy consumption countries are selected as the sample for comparison. The x-axis and the y-axis represent the year and roadway mileage in kilometers, respectively. The z-axis represents the road transport SO_2_ emissions.

Compared with other countries, as illustrated in Figs. 9, 10, this study facilitates a better understanding of China’s FFV development and the level of its related emissions, including both CO_2_ emissions and SO_2_ emissions. In general, the development and expansion of road construction represented by the total length of roadway mileage show different trends in different countries. The faster the construction and expansion of a country’s roadway developed, the higher the road transport SO_2_ emissions generated. In addition, there is a positive relationship between economic development and road transport CO_2_ emissions as well as SO_2_ emissions in developing countries, while this relationship reverses in developed countries or regions.

As shown in Fig. 9, a panoramic figure of the comparison of road transport CO_2_ emissions generated by fossil fuel vehicles and economic development is compared among China, the United States, the European Union, Japan, and India. These five countries or regions are the top five largest energy consumption and economic development countries in the world. It is apparent that the United States’s total road transport CO_2_ emissions are the highest among other countries, which is closely connected to the idea of the United States as a country on wheels. This is partly because China and India are developing countries and possess lower levels of private cars compared to the United States and the European Union. China’s road transport CO_2_ emissions have exceeded those of the EU since 2015. The projection of the y-axis and z-axis represents the connection between economic development and road transport CO_2_ emissions. India has the highest growth rate in road transport CO_2_ emissions along with economic development, and China has the second highest growth rate. Given the rapid development in economic growth in China and India and their vast total population, it can be inferred that the total road transport CO_2_ emissions will grow rapidly. Although road transport only ranks second in each country’s total CO_2_ emissions, phasing out fossil fuel vehicles is important for the heading carbon neutrality target for each country.

In Fig. 10, a panoramic figure of the comparison of road transport SO_2_ emissions generated by fossil fuel vehicles and roadway mileage development is compared among China, the United States, Japan, and India. These four countries have comparatively large energy consumption and impact other countries worldwide. It is apparent that the United State’s total road transport SO_2_ emissions are the highest among other countries, which is closely connected to the United States possessing the largest amount of private car ownership. Currently, there is a shortage of rational indicators for SO_2_ emissions intensity generated by road transport energy. The projection of the y-axis and z-axis represents the connection between roadway development and road transport SO_2_ emissions. Along with the increase in roadway mileage, China’s total SO_2_ emissions generated by road transport have the fastest growth rate, and India has the second fastest growth rate. This is connected to rapid economic growth and the vast population. Japan’s road mileage stays at almost the same level among all these years because of its constrained land area. However, the total road transport SO_2_ emissions are in a growth trend, which indicates that Japan’s road transport fuel is growing too, while the United States has a decreasing trend. In fact, road transport SO_2_ emissions are generated only when fossil fuel vehicles are operating on public roads, which has a deep impact on the environmental quality of areas with high population density, such as urban-based areas. Therefore, from the perspective of reducing SO_2_ emissions in densely populated urban-based areas and improving urban environmental quality, compared with the United States and other developed countries with high car ownership per thousand people, phasing out and banning the sale of fossil fuel vehicles will eventually have a great internal driving force even in most developing countries, such as China and India, which have lower car ownership per thousand people.

## Discussion

The greater ambition of national independent donation (NDC) to control the temperature rise within 1.5 °C worldwide, called for by COP26, will lead to more in-depth discussions on carbon emission reduction control. The banning sales of fossil fuel vehicles involve reducing nearly a quarter of global carbon dioxide emissions and air pollution in densely populated areas. Taking China as an example, this step will be helpful for achieving the requirements of 30–60 dual carbon targets and alleviating the shortage of transportation fuels.

Based on the forecasting results of the Markovian model, the results show an unusual decreasing trend in the rotation volume of both freight and passengers in road transport and a significant increase in railways, indicating that people prefer to avoid road traffic and choose railways instead. This is also a representation of the new policy launched in China’s fourteen-fifth planning regulation about road transition to railway and waterway, which reflects the effectiveness of policy instruments in the transformation of transportation mode and road transportation energy structure. In addition, unexpected events such as COVID-19 have an influential impact on transportation demand.

The results of the regional (30 cities and provinces) carbon intensity of the transportation sector when using the added value of the transportation sector of each city or province as a denominator show that Yunnan, Inner Mongolia, and Ningxia had the highest CEI values from 2003 to 2019. Furthermore, when the GDP of each city or province is used as the denominator, Yunnan, Xinjiang, and Guangxi are the top three provinces and cities with the highest CEI value in the transportation sector. This indicates significant heterogeneity among different regions, similar to other countries with multiple regions and vast territories. The urban and regional carbon intensity of the transportation sector has a high connection with the freight car possession volume.

In the future, the long-term fluctuation of the COVID-19 epidemic and the overall trend of economic growth after the epidemic will coexist for a long time and result in a strong demand for transportation. The demand shares of FFVs and NEVs, respectively show a downward and upward trend overall. Among the new energy vehicles, the demand for BEVs and PHEVs is growing steadily, while the share of FCVs accounted for is too small to play an important role in vehicle transformation in the next 40 years. By encouraging vehicle substitution through appropriate policies, the possible fluctuations of this dramatic carbon emission reduction strategy of the transportation sector can be smoothed under the condition of meeting the mobility needs of residents.

In fact, aside from reducing CO_2_ emissions to combat global climate change, reducing the impact of sulfur-containing automobile exhaust on local residents who live in densely populated urban areas and alleviating the tense supply–demand relationship of fuel oil in the international oil market should be a significant driving force in the near future. Based on the analysis through the theoretical model, different policy instruments could promote the transition to fossil fuel vehicles. A comparison of four different policy scenarios at their steady state indicates that all of the climate policies dampen economic development compared to the BAU scenario. Furthermore, we find that an energy tax can alleviate exogenous shocks’ impact on the economy by decreasing the consumption of energy and road transport. For countries with vast territories, there is regional heterogeneity in both economic development and the level of fossil fuel vehicle ownership. Hence, variation among cities and regions should be treated as an essential factor during the process of policy designation and implementation, and treating different cities and regions differently, such as phased, differentiated, and gradually implemented policies and measures, could profit the local economy to cope with unexpected shocks and enhance economic resilience. With economic growth, the vehicle ownership of developing countries such as China and India are bound to increase greatly. With reference to the situation of developed countries and regions such as the United States, the European Union, and Japan, we should conduct comprehensive research, carry out a transformation layout in advance and formulate corresponding policies and measures in combination with our own actual situation, which can promote the great changes in the transportation field of phasing out fossil fuel vehicles.

In the future, the policy of phasing out fossil fuel vehicles and banning their sale can eventually contribute to emissions mitigation and reduction in fossil fuel consumption. The energy consumption corresponding to the reduced emissions accounts for 10% of the total energy, which is equivalent to carbon dioxide emission reduction (~1 billion tons) and sulfur dioxide emission reduction in central urban areas (~3000 tons). This is of significant importance to improve the urban atmospheric environment and benefit the health of residents. In addition, the oil supply–demand of 400–600 million tons for road transportation fuel can be alleviated, and the positive effect of phasing out traditional fossil energy vehicles can be fully displayed.

## Methods

### Provincial carbon emission intensity and sulfur dioxide intensity of road transport

Since the carbon emission intensity (CEI) (defined as the ratio of carbon emissions to GDP) is regarded as one of the important indicators in carbon emissions reduction by the Chinese government, the CEI of road transport is calculated using the following method. This paper employs panel data of 30 Chinese provinces and municipalities during the period from 2003 to 2019 to consider the accessibility and comprehensiveness of the datasets, which can be defined using both Eq. (12) and Eq. (13).

12 $${\mathrm{CEI}}_{i,t} = {\mathrm{EM}}_{i,t}^c/{\mathrm{TSOV}}_{i,t}$$

13 $${\mathrm{CEI}}^G_{i,t} = {\mathrm{EM}}_{i,t}^c/{\mathrm{GDP}}_{i,t}$$

Here, CEI denotes the carbon emission intensity in the transportation sector, EM^c^ represents the total CO_2_ emissions of the transportation sector, and TSOV stands for the GDP of the transportation sector, which can be obtained from Chinese statistical yearbooks at the provincial level. The subscript *i* and *t* represent the ith province or city and t year, respectively.

Considering the characteristics of the transportation sector, the calculation is divided into two parts: domestic carbon emissions and net import carbon emissions. Domestic carbon emissions are emissions directly generated by vehicles produced by domestic enterprises. The net import carbon emissions are emissions generated by net imports of vehicles. Domestic carbon emissions in the transportation sector can be calculated using the following equation. The energy used by the transport sector is separated into nine types, including coal, coke, crude oil, fuel oil, gasoline, diesel, natural gas, and electricity. The CO_2_ emissions can be calculated as follows.

14 $${\mathrm{CE}}_t = \mathop {\sum}\limits_{i = 1}^n {\zeta _iE_{i,t} \times \frac{{44}}{{12}}}$$

where CE_*t*_ denotes the total CO_2_ emissions of the transport sector at time *t*. Subscript *i* represents the *i*th type of on-road fuel. *E*_*i,t*_ is the energy consumption of the *i*th type of on-road fuel in year *t*. *ζ*_*i*_ represents the carbon emission factor of the ith type of on-road fuel.

In addition, phasing out conventional fossil energy vehicles can alleviate the pressure of energy savings and air pollution. Using the sulfur intensity as one of the measurements of SO_2_ emissions of the transportation sector, the calculation method can be expressed as follows, where *ϕ*^*s*^ denotes the sulfur content of oil with the unit of μg per gram.

15 $${\mathrm{SO}}_{2,i} = 10^{ - 6}\phi ^sE_{i,t} \times \frac{{64}}{{32}}$$

### The Markov-chain model and vehicle structural prediction by type

The Markov-chain model denotes a stochastic process. A stochastic process is a family of random variables {*X*(*t*), *t* ∈ *T*} defined on a given probability space *S*, indexed by the parameter *t*, where *t* belongs to an index set *T*. Specifically, a transfer process is called a Markov-chain when the conditional probability satisfies Eq. (16):

16 $$P\left\{ {X_{n + 1} = s_{n + 1}|X_0 = s_0,X_1 = s_1,X_2 = s_2, \ldots ,X_n = s_n} \right\} = P\left\{ {X_{n + 1} = s_{n + 1}|X_n = s_n} \right\}$$

where *S* is the collection of all possible values that the random variables of the stochastic process may assume. If $P\left\{ {X_{n + 1} = s_{n + 1}|X_0 = s_0,X_1 = s_1,X_2 = s_2, \ldots ,X_n = s_n} \right\} > 0$ is applicable for any positive integer *n* ∈ *N* and any state *s*_1_,*s*_2_,…,*s*_*n*_∈*S*, the values assumed by a random variable *X*_*n*_ are called “states”. Equation (3) denotes that the state at moment *n* + 1 is dependent on only the state at moment *n*. It is a significant feature of a Markov process that the future state is merely associated with the current state and does not rely on the previous state^41^. This characteristic is known as the “nonaftereffect” property^42^ or “memoryless” property. For all *i,j*∈*S*_*n*_, the Markov-chain one-step transition probability matrix is defined in Eq. (17), which denotes a state transition from moment n to moment *n* + 1.

17 $$P\left\{ {X_{n + 1} = j|X_n = i} \right\} \buildrel \Delta \over = p_{ij}\left( n \right)\left( {p_{ij} \ge 0;\mathop {\sum}\nolimits_{j \in S} {p_{ij}\left( n \right) = 1} } \right)$$

When the transition probability is independent of *n*, the Markov-chain becomes a homogeneous Markov-chain, which is the most commonly used random process. Hereafter, the one-step transition probability matrix is defined in Eq. (18), and *p*_*ij*_(*n*) becomes *p*_*ij*_.

18 $$P = (p_{ij}) = \left[ {\begin{array}{*{20}{c}} {p_{11}} & {p_{12}} & \ldots & {p_{1n}} \\ {p_{21}} & {p_{22}} & \ldots & {p_{2n}} \\ \vdots & \vdots & {} & \vdots \\ {p_{n1}} & {p_{n2}} & \ldots & {p_{nn}} \end{array}} \right]$$

Under this assumption, the *k* step transition probability is calculated by Eq. (19) as follows:

19 $$P_{ij}^{\left( k \right)} = P\left\{ {M_{n + k} = j|M_n = i} \right\}$$

According to the assumption of the Chapman–Kolmogorov equation, if the initial probability distribution vector is *P*_0_, the probability distribution of the state after *k* periods can be calculated by Eq. (20). For more information about Chapman–Kolmogorov assumption, please refer to the supplementary notes in the Supplementary Information file

20 $$P\left( k \right) = P_0P^k$$

The variation in the energy consumption structure is identified as a stochastic process^41,43^. A Markov-chain model is employed to forecast vehicle consumption by powertrains in China from 2020 to 2060, with the initial input data period from 2011 to 2019. Assume the state space S contains all the historical vehicle consumption state vectors S_i_, which can be described as $S_i = \{ {S_{ff}\left( i \right),S_p^{nev}\left( i \right),S_{hyb}^{nev}\left( i \right),S_{fb}^{nev}\left( i \right)} \}$. The four elements $S_{ff}\left( i \right),S_p^{nev}\left( i \right),S_{hyb}^{nev}\left( i \right)$ and $S_{fb}^{nev}\left( i \right)$ in *S*_*i*_ represent the initial state for each type of powertrain, namely, fossil fuel vehicles, pure electric vehicles, hybrid plug-in electric vehicles, and fuel battery electric vehicles, respectively, and take each type of powertrain consumption ratio to the total vehicle consumption as the measurement index.

The calculations of the transition probability matrix are the most critical procedure during the Markov-based model prediction process^41,43^. The position transition matrix comprises transition probabilities that each type of energy’s share in the primary energy consumption changes or the status quo. The one-step transition probability matrix is shown in Eq. (21):

21 $$P_i{{{\mathrm{ = }}}}\left[ {\begin{array}{*{20}{c}} {p_{ff \to ff}\left( i \right)} & {p_{ff \to nevp}\left( i \right)} & {p_{ff \to nevhyb}\left( i \right)} & {p_{ff \to nevfb}\left( i \right)} \\ {p_{nevp \to ff}\left( i \right)} & {p_{nevp \to nevp}\left( i \right)} & {p_{nevp \to nevhyb}\left( i \right)} & {p_{nevp \to nevfb}\left( i \right)} \\ {p_{nevhyb \to ff}\left( i \right)} & {p_{nevhyb \to nevp}\left( i \right)} & {p_{nevhyb \to nevhyb}\left( i \right)} & {p_{nevhyb \to nevfb}\left( i \right)} \\ {p_{nevfb \to ff}\left( i \right)} & {p_{nevfb \to nevp}\left( i \right)} & {p_{nevfb \to nevhyb}\left( i \right)} & {p_{nevfb \to nevfb}\left( i \right)} \end{array}} \right]$$

where the main diagonal elements denote the original share probability of the four types of vehicle consumption, the row elements indicate the conversion probability for each subcategory, and the column elements represent the switching probability from other subcategories.

If available, vehicle and energy consumption data in this paper are from the Chinese Statistical Yearbook (CSY). Based on historical data from 2003 to 2019, the prediction of vehicle consumption from 2020 to 2060 is performed in three steps. First, suppose the initial state of the vehicle consumption structure by their powertrains is in 2011. Second, the one-step transition probability matrix^44^ is calculated annually with 1 year as the time interval. Third, the average probability transition matrix is calculated. The average transition matrix can be obtained by means of Eq. (22).

22 $$P = \left[ {\mathop {{\Pi}}\limits_{i = 1}^n P\left( i \right)} \right]^{\frac{1}{n}}$$

The main diagonal element equals 1 when a type of energy has a higher ratio of primary energy consumption at time *t* + 1 than at time *t*. In other words, the conversion probability of this type of energy is 1.

### Dynamic stochastic general equilibrium model

To evaluate how different policy instruments affect the economy and energy-related emissions, we first consider a determined economy. Consider a simple Robinson Crusoe economy with reference to Fischer and Springborn^40^. Let *C* be the consumption good, *K* be the capital stock, *H* be the labor supply, *l* be the leisure time, and *M* be the polluting intermediate good. In this context, *M* includes CO_2_ and SO_2_ emissions. The representative agent obtains utility in period t equal to *U*(*C*_*t*_, 1 − *H*_*t*_) from consuming goods and spending leisure time. Therefore, the utility function can be represented as follows.

23 $$U\left( {C_t,1 - H_t} \right) = \ln C_t + \eta \ln \left( {1 - H_t} \right)$$

We take the format of logarithm with additive with the reference to Hansen^45^. The total output *Y* is a Cobb–Douglas function of capital, labor supply, and pollution intermediate inputs (including road transport emissions), adjusted by a productivity factor *Z* with an expected value of 1, where the total output can be expressed as follows.

24 $$Y_t = f\left( {K_t,M_t,H_t} \right) = Z_tK_t^\alpha M_t^\gamma H_t^{1 - \alpha - \gamma }$$

We assume that the capital accumulation follows the following dynamic rule as in *K*_*t*+1_ = (1 − *δ*)*K*_*t*_ + *I*_*t*_, where *I*_*t*_ denotes the gross investment and *δ* is the depreciated rate. This indicates that during each period, capital depreciates at rate *δ* and is augmented by gross investment. The total output is allocated among consumption, investment, and pollution intermediate goods (*C* + *I* + *M* ≤ *Y*), which is an important budget constraint in this model. Following the assumptions in Fischer and Springborn^40^, the level of polluting intermediate inputs and the level of emissions are used interchangeably in the following analysis. The emissions constraint represents *M* ≤ *A*_*t*_(*Y*), where *A*(•) is a function of permit allocation and varies over time. The Lagrangian function for maximizing the expected lifetime utility is as follows.

25 $${\mathrm{L}} = \max E\mathop {\sum}\nolimits_{t = 0}^\infty {\left\{ \begin{array}{l}\beta ^t\left[ {\ln C_t + \eta \ln \left( {1 - H_t} \right)} \right] + \lambda _t\left[ {Z_tK_t^\alpha M_t^\gamma H_t^{1 - \alpha - \gamma } - C_t - M_t - K_{t + 1} + \left( {1 - \delta } \right)K_t} \right] + \\ \psi _t\left[ {A_t\left( {Z_tK_t^\alpha M_t^\gamma H_t^{1 - \alpha - \gamma }} \right) - M_t} \right]\end{array} \right\}}$$

The representative agent is assumed to make a decision in period *t* based on all the information available at time *t*, which indicates that there is no asymmetry in the information accessibility. In Eq. (25), *β* represents the discount factor, *λ*_*t*_ is the shadow price of the national income identity, and *ψ*_*t*_ is the shadow value of the emissions constraint. The maximization problem actually depicts the discounted sum of utility along with the infinite lifetime of the representative agent. Under this determined economy scenario, any additional revenue generated by the policy is conserved within the system as lump-sum transfers. According to the Lagrangian function, we can obtain the first-order conditions of each variable as follows:

26 $$C_t:\quad \frac{1}{{C_t}} = \lambda _t$$

27 $$K_t:\quad \alpha Z_tK_t^{\alpha - 1}M_t^\gamma H_t^{1 - \alpha - \gamma }\left( {1 + \hat \phi A_{t,Y}} \right) = \beta E_t\left( {\frac{{\lambda _{t - 1}}}{{\lambda _t}}} \right) - (1 - \delta )$$

28 $$M_t:\quad \gamma Z_tK_t^\alpha M_t^{\gamma - 1}H_t^{1 - \alpha - \gamma }\left( {1 + \hat \phi _tA_{t,Y}} \right) = 1 + \hat \phi _t$$

29 $$H_t:\quad \left( {1 - \alpha - \gamma } \right)Z_tK_t^\alpha M_t^\gamma H_t^{ - \alpha - \gamma }\left( {1 + \hat \phi _tA_{t,Y}} \right) = \frac{{\eta C_t}}{{1 - H_t}}$$

30 $$\lambda _t:\quad Z_tK_t^\alpha M_t^\gamma H_t^{1 - \alpha - \gamma } = K_{t + 1} - \left( {1 - \delta } \right)K_t + C_t + M_t$$

31 $$\psi _t:\quad M_t = A_t\left( {Y_t} \right)$$

Where *A*_*t,Y*_ represents a first-order derivative of *A*_*t*_ with respect to *Y*_*t*_.

Based on the DSGE framework above, we further introduce return on capital, labor income, and energy consumption instead of emission intermediates to evaluate the reaction of energy consumption to exogenous shocks and energy price fluctuations.

The representative enterprises wish to maximize their net profit, i.e., the total revenue from output minus the production cost.

32 $$\max Y_t - w_tH_t - r_tK_t - P_t^eE_t$$

where the price of final products is normalized to one, Y_t_ represents the total output, and *w*_*t* t_ and *r*_*t*_ denote the wage rate and the capital return, respectively. Under a complete competition factor market, the capital return equals the interest rate. $P_t^e$ is the energy price.

33 $${\mathrm{EM}}_t^c = \left( {1 - \xi _t} \right)\phi _t^c\psi E_t$$

34 $${\mathrm{EM}}_t^s = \left( {1 - \xi _t} \right)\phi _t^s\psi E_t \times 10^{ - 6} \times \frac{{64}}{{32}}$$

where superscripts *s* and *c* denote emissions of sulfur dioxide and carbon dioxide, respectively, E_t_ denotes the total consumption of energy, *ψ* is the share of on-road fuel to the total energy consumption, and *ξ*_*t*_ represents the share of nonfossil fuel consumption to the total energy use by vehicles at time t. Assume that energy price $P_t^e$ follows an AR (1) process:

35 $$P_t^e = \rho P_{t - 1}^e + \varepsilon _t$$

All the parameters used in the DSGE framework are calibrated and the corresponding values are provided in Supplementary Table 5. Besides, all the abbreviations used in this study are displayed with their meaning in Supplementary Table 6.

### Reporting summary

Further information on research design is available in the Nature Research Reporting Summary linked to this article.

## Supplementary information

Supplementary Material

Reporting Summary

## Data Availability

The datasets generated during and/or analyzed during the current study are available from the corresponding author upon reasonable request. Part of the data were publicly dataset as follows, the CEADs dataset are available at https://www.ceads.net/. The Chinese energy statistical yearbook is proprietary and is thus not freely available. The provincial and city-level population data were available at https://data.stats.gov.cn/easyquery.htm?cn=E0103. The road mileage data of the United States is from the US Department of transportation, available at http://www.fhwa.dot.gov/policyinformation/statistics.cfm. The road mileage data of India is from the Ministry of Road Transport & Highways, Government of India, which is available at https://morth.nic.in/sites/default/files/Annual%20Report%20-%202021%20%28English%29_compressed.pdf. Oil product’s final consumption by sector is from the IEA World Energy Balances available at https://www.iea.org/data-and-statistics/data-product/world-energy-statistics-and-balances.

## Code availability

The code aggregated and/or analyzed during the current study are available from the corresponding author upon reasonable request.
