# Supplementary material for: Promoting economic and environmental resilience in the post-COVID-19 era through the city and regional on-road fuel sustainability development
Source: NPJ Urban Sustain. 2022 Dec 15;2(1):33. doi: 10.1038/s42949-022-00078-6 (PMC9751518; doi:10.1038/s42949-022-00078-6)
Supplement: Supplementary file 1 — Supplementary Material [file 42949_2022_78_MOESM1_ESM.pdf]

# Supplementary Information

# Promoting economic and environmental resilience in the post-COVID-19 era through the city and regional on-road fuel sustainability development

Chuxiao Yang [1,2], Haitao Wu[1,2], Yunxia Guo[1,2], Yu Hao [1,2,3,4,5, *], Zhaohua Wang [1,2,4,6 **]

1 School of Management and Economics, Beijing Institute of Technology, Beijing, China.
2 Center for Energy and Environmental Policy Research, Beijing Institute of Technology, Beijing, China.
3 Yangtze Delta Region Academy of Beijing Institute of Technology, Jiaxing, China.
4 Sustainable Development Research Institute for Economy and Society of Beijing, Beijing, China.
5 Beijing Key Lab of Energy Economics and Environmental Management, Beijing, China.
6 Research Center for Sustainable Development & Intelligent Decision, Beijing Institute of Technology, Beijing, China
*, **: Corresponding authors haoyuking@gmail.com, haoyuking@bit.edu.cn (Yu Hao); wangzhaohua@bit.edu.cn (Zhaohua Wang);

## Supplementary Tables

**Supplementary Table 1 | Corresponding province name in Fig. 3.** Name of cities or province was plotted as sequential numbers in Fig. 3. This table provides each city and province's name accordingly. The order is in ascending according to each province's transportation sector carbon intensity with the GDP of corresponding province or city as denominator.

| Province | Number | Province | Number |
|---|---|---|---|
| Hebei | 1 | Shanxi | 16 |
| Beijing | 2 | Chongqing | 17 |
| Shanghai | 3 | Innermongolia | 18 |
| Tianjin | 4 | Shaanxi | 19 |
| Anhui | 5 | Guangdong | 20 |
| Henan | 6 | Gansu | 21 |
| Fujian | 7 | Hubei | 22 |
| Hainan | 8 | Ningxia | 23 |
| Jiangsu | 9 | Qinghai | 24 |
| Jilin | 10 | Heilongjiang | 25 |
| Shandong | 11 | Liaoning | 26 |
| Zhejiang | 12 | Guizhou | 27 |
| Hunan | 13 | Guangxi | 28 |
| Jiangxi | 14 | Xinjiang | 29 |
| Sichuan | 15 | Yunnan | 30 |

**Supplementary Table 2 | Corresponding province name in Fig.4.** Name of cities or province was plotted as sequential numbers in Fig. 4. This table provides each city or province's name accordingly. The order of provinces is in ascending according to each province or city's average transportation sector carbon emission intensity with the GDP of the transport sector as the denominator.

| Province | Number | Province | Number |
|---|---|---|---|
| Beijing | 1 | Chongqing | 16 |
| Shanghai | 2 | Heilongjiang | 17 |
| Jiangsu | 3 | Jiangxi | 18 |
| Zhejiang | 4 | Shaanxi | 19 |
| Henan | 5 | Hubei | 20 |
| Fujian | 6 | Shanxi | 21 |
| Hainan | 7 | Qinghai | 22 |
| Tianjin | 8 | Gansu | 23 |
| Sichuan | 9 | Guangxi | 24 |
| Hebei | 10 | Xinjiang | 25 |
| Anhui | 11 | Guizhou | 26 |
| Guangdong | 12 | Liaoning | 27 |
| Jilin | 13 | Yunnan | 28 |
| Shandong | 14 | Inner Mongolia | 29 |
| Hunan | 15 | Ningxia | 30 |

**Supplementary Table 3 | Corresponding province name in Fig.7.** Name of cities or province was plotted as sequential numbers in Fig. 7. This table provides each city or province's name accordingly. The order of cities and provinces are the same as original data in the CEADs.

| **Province** | **Number** | **Province** | **Number** |
|---|---|---|---|
| Shanghai | 1 | Jiangxi | 16 |
| Yunnan | 2 | Hebei | 17 |
| Innner Mongolia | 3 | Henan | 18 |
| Beijing | 4 | Zhejiang | 19 |
| Jilin | 5 | Hainan | 20 |
| Sichuan | 6 | Hubei | 21 |
| Tianjin | 7 | Hunan | 22 |
| Ningxia | 8 | Gansu | 23 |
| Anhui | 9 | Fujian | 24 |
| Shandong | 10 | Guizhou | 25 |
| Shanxi | 11 | Liaoning | 26 |
| Guangdong | 12 | Chongqing] | 27 |
| Guangxi | 13 | Shaanxi | 28 |
| Xinjiang | 14 | Qinghai | 29 |
| Jiangsu | 15 | Heilongjiang | 30 |

**Supplementary Table 4 | Steady state expressions comparison**

| | No Policy | Emission intensity | Emission cap | Emission Tax |
|---|---|---|---|---|
| k | $\hat{\kappa}\alpha$ | $\frac{\hat{\kappa}\alpha(1-\mu)}{1-\gamma}$ | $\hat{\kappa}\alpha$ | $\hat{\kappa}\alpha$ |
| c | $1-\gamma-\delta\hat{\kappa}\alpha$ | $1-\mu-\delta\frac{\hat{\kappa}\alpha(1-\mu)}{1-\gamma}$ | $1-\frac{\gamma}{1+\hat{\phi}}-\delta\hat{\kappa}\alpha$ | $1-\gamma/(1+\tau)-\delta\hat{\kappa}\alpha$ |
| d | $\frac{1-\alpha-\gamma}{\eta(1-\gamma-\delta\hat{\kappa}\alpha)}$ | $\frac{1-\alpha-\gamma}{\eta(1-\gamma-\delta\hat{\kappa}\alpha)}$ | $\frac{1-\alpha-\gamma}{\eta c}$ | $\frac{1-\alpha-\gamma}{\eta c}$ |
| m | $\gamma$ | $\frac{\gamma(1+\hat{\phi}\mu)}{1+\hat{\phi}}$ | $\frac{\gamma}{1+\hat{\phi}}=\frac{\bar{M}}{\bar{Y}}$ | $\frac{\gamma}{1+\tau}=\frac{\bar{M}}{\bar{Y}}$ |

**Supplementary Table 5 | Summary of simulation parameter values**

| Parameter | Description | Value | Literature resources |
|---|---|---|---|
| $\gamma$ | Cobb-Douglas parameter for H | 0.349 | Sun & Jiang (2012) |
| $\alpha$ | Cobb-Douglas parameter for K | 0.493 | Fan et al. (2016) |
| $\eta$ | Utility parameter | 0.77 | Sun & Jiang (2012) |
| $\beta$ | Discount factor | 0.985 | He et al. (2017) |
| $\delta$ | Depreciation rate | 0.025 | Bai et al. (2006) |
| $\rho_z$ | Shock persistence parameter | 0.95 | Fischer & Heutel (2013) |

**Supplementary Table 6 | Abbreviation notation list**

| Abbreviations | Meaning |
|---|---|
| BEVs | Battery electrical vehicles |
| BP | BP p.l.c. |
| CEADs | Carbon Emission Accounts & Datasets for emerging economies |
| CEI | Carbon emission intensity |
| CBDR | Common but differentiated responsibilities |
| DSGE | Dynamic stochastic general equilibrium |
| FCVs | Fossil cell vehicles |
| FFVs | Fossil fuel vehicles |
| IEA | International Energy Agency |
| NEVs | New energy vehicles |
| PHEVs | Plug-in hybrid electrical vehicles |
| TWC | Three Way Catalytic Converter |

**Supplementary Notes:**

When the stochastic process under consideration is Markovian, the Chapman-Kolmogorov equation is equivalent to an identity on transition densities. When the probability distribution on the state space of a Markov chain is discrete and the Markov chain is homogeneous, the Chapman-Kolmogorov equations can be expressed in terms of matrix multiplication; thus, P(t+s)=P(t)P(s), where P(t) is the transition matrix of jump t and X_t is the state at time t. (For more information, please refer to https://en.wikipedia.org/wiki/Chapman%E2%80%93Kolmogorov_equation and Chapman-Kolmogorov Equation - an overview | ScienceDirect Topics (accessed on May 4th, 2021).)

**Supplementary References:**


Bai, C. E., Hsieh, C. T., & Qian, Y. (2006). The return to capital in China. Brookings Papers on Economic Activity, (2), 61-102.

Fan, Q., Zhou, X., & Zhang, T. (2016). Externalities of dynamic environmental taxation, paths of accumulative pollution and long-term economic growth. Economic Research Journal, 8, 116-128.

Fischer, C., & Heutel, G. (2013). Environmental macroeconomics: Environmental policy, business cycles, and directed technical change. Annu. Rev. Resour. Econ., 5(1), 197-210.

He, Q., Liu, F., Qian, Z., & Chong, T. T. L. (2017). Housing prices and business cycle in China: A DSGE analysis. International Review of Economics & Finance, 52, 246-256.

Sun, N. H., & Jiang, X. D. (2012). Energy Price and Macro-Economy: Dynamic Model and Calibration. NaiKai Economic Studies, 2, 20-32.
