# Supplementary material for: Promoting economic and environmental resilience in the post-COVID-19 era through the city and regional on-road fuel sustainability development
Source: NPJ Urban Sustain. 2022 Dec 15;2(1):33. doi: 10.1038/s42949-022-00078-6 (PMC9751518; doi:10.1038/s42949-022-00078-6)
Supplement: Supplementary file 2 — Reporting Summary [file 42949_2022_78_MOESM2_ESM.pdf]

nature portfolio

Corresponding author(s): Yu Hao

Last updated by author(s): Oct 14, 2022

Reporting Summary

Nature Portfolio wishes to improve the reproducibility of the work that we publish. This form provides structure for consistency and transparency in reporting. For further information on Nature Portfolio policies, see our Editorial Policies and the Editorial Policy Checklist.

Statistics

For all statistical analyses, confirm that the following items are present in the figure legend, table legend, main text, or Methods section.

n/a	Confirmed	
☐	☒	The exact sample size (*n*) for each experimental group/condition, given as a discrete number and unit of measurement
☐	☒	A statement on whether measurements were taken from distinct samples or whether the same sample was measured repeatedly
☒	☐	The statistical test(s) used AND whether they are one- or two-sided<br>*Only common tests should be described solely by name; describe more complex techniques in the Methods section.*
☒	☐	A description of all covariates tested
☐	☒	A description of any assumptions or corrections, such as tests of normality and adjustment for multiple comparisons
☒	☐	A full description of the statistical parameters including central tendency (e.g. means) or other basic estimates (e.g. regression coefficient) AND variation (e.g. standard deviation) or associated estimates of uncertainty (e.g. confidence intervals)
☒	☐	For null hypothesis testing, the test statistic (e.g. *F*, *t*, *r*) with confidence intervals, effect sizes, degrees of freedom and *P* value noted<br>*Give P values as exact values whenever suitable.*
☒	☐	For Bayesian analysis, information on the choice of priors and Markov chain Monte Carlo settings
☒	☐	For hierarchical and complex designs, identification of the appropriate level for tests and full reporting of outcomes
☒	☐	Estimates of effect sizes (e.g. Cohen's *d*, Pearson's *r*), indicating how they were calculated

Our web collection on statistics for biologists contains articles on many of the points above.

Software and code

Policy information about availability of computer code

Data collection	No software was used for data collection
Data analysis	We used both Matlab 2021a and Excel 2019 to generate the results showed in Fig.1-Fig.4. We used software R version x64 4.1.1 to generate the results showed in Figure 5.We used Matlab 2021a to generate the results showed in Fig.6 and Fig.7. We used Matlab 2021a and dynare software version 4.5.11 to generate the results showed in Fig.8 . Dynare is a software platform for handling a wide class of economic models, in particular dynamic stochastic general equilibrium (DSGE). For more information, please refer to Stéphane Adjemian, Houtan Bastani, Michel Juillard, Frédéric Karamé, Ferhat Mihoubi, Willi Mutschler, Johannes Pfeifer, Marco Ratto, Normann Rion and Sébastien Villemot (2022), "Dynare: Reference Manual, Version 5," Dynare Working Papers, 72, CEPREMAP. We used origin version 2022 SR1 to generate the results showed in Fig.9 and Fig.10.

For manuscripts utilizing custom algorithms or software that are central to the research but not yet described in published literature, software must be made available to editors and reviewers. We strongly encourage code deposition in a community repository (e.g. GitHub). See the Nature Portfolio guidelines for submitting code & software for further information.

Data

Policy information about availability of data

All manuscripts must include a data availability statement. This statement should provide the following information, where applicable:

- Accession codes, unique identifiers, or web links for publicly available datasets
- A description of any restrictions on data availability
- For clinical datasets or third party data, please ensure that the statement adheres to our policy

The datasets aggregated and/or analyzed during the current study are available from the corresponding author on reasonable request. The CEADs dataset are available at https://www.ceads.net/. The Chinese energy statistical yearbook is proprietary and is thus not freely available. The provincial and city level population data is available at https://data.stats.gov.cn/easyquery.htm?cn=E0103. The road mileage data of U.S. is from U.S Department of transportation available at http://www.fhwa.dot.gov/policyinformation/statistics.cfm. The road mileage data of India is from the ministry of road transport & highways, government of India, which is available at https://morth.nic.in/sites/default/files/Annual%20Report%20-%202021%20%28English%29_compressed.pdf. Oil products final consumption by sector is from the IEA World Energy Balances available at https://www.iea.org/data-and-statistics/data-product/world-energy-statistics-and-balances.

Human research participants

Policy information about studies involving human research participants and Sex and Gender in Research.

Reporting on sex and gender	This study didn't involved with any human research participants.
Population characteristics	See above.
Recruitment	See above.
Ethics oversight	See above.

Note that full information on the approval of the study protocol must also be provided in the manuscript.

Field-specific reporting

Please select the one below that is the best fit for your research. If you are not sure, read the appropriate sections before making your selection.

☐ Life sciences ☒ Behavioural & social sciences ☐ Ecological, evolutionary & environmental sciences

For a reference copy of the document with all sections, see nature.com/documents/nr-reporting-summary-flat.pdf

Behavioural & social sciences study design

All studies must disclose on these points even when the disclosure is negative.

Study description	Data applied in this study are quantitative, mostly time series and panel data.
Research sample	This study is mainly involving with existing data. The data sets aggregated and/or analyzed during the current study are consisting of both publicly database and payed database. All the database involved in this study are as follows. The CEADs dataset are available at https://www.ceads.net/. The Chinese energy statistical yearbook is proprietary and is thus not freely available. The provincial and city level population data is available at https://data.stats.gov.cn/easyquery.htm?cn=E0103. The road mileage data of U.S. is from U.S Department of transportation available at http://www.fhwa.dot.gov/policyinformation/statistics.cfm. The road mileage data of India is from the ministry of road transport & highways, government of India, which is available at https://morth.nic.in/sites/default/files/Annual%20Report%20-%202021%20%28English%29_compressed.pdf. Oil products final consumption by sector is from the IEA World Energy Balances available at https://www.iea.org/data-and-statistics/data-product/world-energy-statistics-and-balances.
Sampling strategy	No sample-size calculation was performed. The sample was chosen consistently for data accessibility and long enough to represent a good result.
Data collection	The original data were collected from both publicly datasets and payed database. All of the collected data were stored in the computer.
Timing	Data span for the CEADs dataset is from 2003 to 2019. Data span for the road mileage for five different countries is from 2010 to 2019. Data span for the IEA energy balance data is from 2009 to 2019.
Data exclusions	No data were excluded from the analysis.
Non-participation	No participants were involved in this study.

Randomization	This study does not include randomized controlled trials, and data were not organized into experimental groups.

Reporting for specific materials, systems and methods

We require information from authors about some types of materials, experimental systems and methods used in many studies. Here, indicate whether each material, system or method listed is relevant to your study. If you are not sure if a list item applies to your research, read the appropriate section before selecting a response.

Materials & experimental systems

n/a	Involved in the study
☒	☐ Antibodies
☒	☐ Eukaryotic cell lines
☒	☐ Palaeontology and archaeology
☒	☐ Animals and other organisms
☒	☐ Clinical data
☒	☐ Dual use research of concern

Methods

n/a	Involved in the study
☒	☐ ChIP-seq
☒	☐ Flow cytometry
☒	☐ MRI-based neuroimaging
